# Preoperative Albumin–Bilirubin (ALBI) Score Is the Strongest Predictor of Mortality After LVAD Implantation

**DOI:** 10.3390/biomedicines13102449

**Published:** 2025-10-08

**Authors:** Tomasz Niklewski, Michał Jurkiewicz, Wioletta Szczurek-Wasilewicz, Bożena Szyguła-Jurkiewicz, Michał Skrzypek, Piotr Przybyłowski, Tomasz Hrapkowicz

**Affiliations:** 1 Department of Cardiac, Vascular and Endovascular Surgery and Transplantology, Silesian Center for Heart Diseases, 41-800 Zabrze, Poland; 2 Student’s Scientific Society, 3rd Department of Cardiology, Faculty of Medical Sciences in Zabrze, Medical University of Silesia, 40-055 Katowice, Poland; 3 Student’s Scientific Association, Department of Cardiac, Vascular and Endovascular Surgery and Transplantology, Faculty of Medical Sciences in Zabrze, Medical University of Silesia, 40-055 Katowice, Poland; 4 2nd Department of Cardiology and Angiology, Silesian Center for Heart Diseases, 41-800 Zabrze, Poland; 5 Department of Pharmacology, Faculty of Medicine, University of Opole, 45-052 Opole, Poland; 6 3rd Department of Cardiology, Faculty of Medical Sciences in Zabrze, Medical University of Silesia, 40-055 Katowice, Poland; 7 Department of Biostatistics, Faculty of Public Health in Bytom, Medical University of Silesia in Katowice, 40-055 Katowice, Poland; 8 Department of Cardiac, Vascular and Endovascular Surgery and Transplantology, Faculty of Medical Sciences in Zabrze, Medical University of Silesia, 40-055 Katowice, Poland

**Keywords:** albumin–bilirubin ratio, heart failure, left ventricular assist device, risk stratification

## Abstract

**Background:** Patients with end-stage heart failure (HF) undergoing left ventricular assist device (LVAD) implantation remain at significant risk of post-implant mortality. Identifying preoperative predictors of adverse outcomes may improve risk stratification. The aim of the study was to search for factors associated with worse prognosis after LVAD implantation during the long-term follow-up. **Methods:** This single-center, retrospective study included 95 patients who underwent HeartMate III LVAD implantation between 2016 and 2024. Indications for implantation included bridge to transplant, bridge to recovery, or destination therapy. Pre-implant clinical data, pharmacological treatment, echocardiographic parameters, and laboratory profiles were collected. Albumin–bilirubin (ALBI) score was calculated as the marker of liver dysfunction. The primary end-point was all-cause mortality during the long-term follow-up. **Results:** The median age was 57.9 years (IQR: 47.2–63.8), and 91.6% were male. During follow-up (835 (250–1973) days), 46 patients (48.4%) died. In multivariable weighted Cox analysis, higher serum creatinine level (HR 3.403 per 1 µmol/L *p* < 0.001), higher ALBI score (HR 4.981 per 1-unit increase; *p* < 0.001) and older age (1.04 per year, *p* < 0.01) remained independent predictors of mortality. **Conclusions:** Among patients undergoing HeartMate III LVAD implantation, higher creatinine concentrations, higher preoperative ALBI score and older age were independently associated with all-cause mortality. These parameters may be useful for risk stratification during long-term follow up.

## 1. Introduction

Despite progress in pharmacological and device-based management of advanced heart failure (HF), it remains a leading cause of morbidity and mortality in developed countries. Due to the persistent shortage of donor organs, only a limited proportion of eligible patients undergo heart transplantation (HT), while mortality on the waiting list continues to rise [1,2]. Therefore, left ventricular assist devices (LVADs) have become an essential component of care for end-stage HF patients, acting as bridge-to-transplant, bridge-to-recovery, or destination therapy [2,3]. The fully magnetically levitated HeartMate III pump reduces device thrombosis and stroke, and markedly improves survival compared with earlier systems [2]; nevertheless, 5-year mortality still approaches 40% in real-world cohorts [4]. These data highlight the need for searching reliable and easily available biomarkers to improve risk stratification and guide LVAD management strategies [5]. In advanced HF, impaired hepatic perfusion and right-sided venous congestion frequently contribute to hepatic dysfunction, which is associated with poor outcomes. Consequently, liver biomarkers have gained attention as potential prognostic tools in the patients with HF [6]. The albumin–bilirubin (ALBI) score, initially developed to quantify liver dysfunction in hepatocellular carcinoma, incorporates two routine laboratory markers—albumin and total bilirubin—and reflects both synthetic and metabolic liver function [7,8]. Several studies have shown that elevated ALBI scores are associated with adverse prognosis in patients with acute and chronic HF [7,9], and that hepatic dysfunction prior to LVAD implantation predicts increased postoperative morbidity and mortality [10,11]. However, the utility of ALBI score for risk stratification after LVAD has not been investigated.

Therefore, the aim of the present study was to evaluate whether the preoperative ALBI score, together with routine clinical and biochemical biomarkers can predict all-cause mortality in advanced HF patients receiving HeartMate III LVAD support.

## 2. Materials and Methods

### 2.1. Study Population

This retrospective single-center study included adult patients with end-stage HF who underwent HeartMate III LVAD implantation between 2016 and 2024 at our institution. All patients were classified as INTERMACS profiles II–III and New York Heart Association (NYHA) class III or IV. Indications for LVAD implantation were bridge to transplant, bridge to recovery, or destination therapy. All included patients were stratified into survivors and non-survivors based on the follow-up data. Patients were followed according to our institutional protocol with regular outpatient visits and clinical assessments. Follow-up data were collected through chart review and the institutional LVAD registry. The primary endpoint was all-cause mortality from LVAD implantation to January 2025.

The study was conducted in accordance with the Declaration of Helsinki. Due to the retrospective nature of the analysis, the requirement for written informed consent was waived.

### 2.2. Clinical and Laboratory Data

Demographic, clinical, echocardiographic, and laboratory data were collected from hospital records immediately before LVAD implantation. Data collected included demographics (age, sex, body mass index), clinical parameters (NYHA classification, presence of ventricular arrhythmias such as non-sustained ventricular tachycardia (nsVT), history of electrical storm, acetylsalicylic acid (ASA) resistance, and comorbidities.

Laboratory assays were conducted using standard automated analyzers. Hematologic parameters were measured with Sysmex XS1000i and XE-2100 counters (Sysmex Corp., Kobe, Japan), and blood chemistries (including creatinine, total bilirubin, albumin, liver enzymes, glucose, and N-terminal pro–B-type natriuretic peptide [NT-proBNP]) were measured using COBAS 6000 analyzer (Roche Diagnostics, Basel, Switzerland). The high-sensitivity C-reactive protein (hs-CRP) level was determined via latex immunoassay (Cobas Integra 70, Roche, Basel, Switzerland). For each patient, we calculated the ALBI score as a quantitative marker of liver function using the published formula.

Platelet function in response to acetylsalicylic acid was evaluated using impedance aggregation in a Multiplate analyzer (Roche Diagnostics, Mannheim, Germany), with a reference range from 745 to 1361 AU × min.

ALBI score was calculated for each patient using the following formula: ALBI = 0.66 × log_10_[total bilirubin (μmol/L)] − 0.0852 × [albumin (g/L)] [7,8].

The CRP-to-bilirubin ratio was determined by dividing the CRP concentration (mg/L) by the total bilirubin (μmol/L).

The relative lymphocyte count (RLC) was calculated as the ratio of lymphocyte and total WBC counts.

### 2.3. Echocardiographic Assessment

Transthoracic echocardiography was performed by two independent experienced cardiologists within 72 h before LVAD implantation as a part of the standard preoperative evaluation. Echocardiographic parameters included measurements of left atrial diameter (LA), right ventricular end-diastolic diameter (RVEDd, in 4-chamber and M-mode), tricuspid annular plane systolic excursion (TAPSE), estimated right ventricular systolic pressure (RVSP), left ventricular end-diastolic diameter (LVEDd), and left ventricular ejection fraction (LVEF).

### 2.4. Pharmacological Treatment and Device Implantation

Patients were treated with standard HF therapy for at least 3 months before implantation in accordance with the guidelines of the European Society of Cardiology in force at the time. Oral anticoagulation with warfarin was initiated early postoperatively, with a target international normalized ratio (INR) of 2.0–3.0. During the transition to therapeutic INR levels, patients received bridging therapy with therapeutic doses of low-molecular-weight heparin (LMWH). In addition to anticoagulation, all patients received antiplatelet therapy. Standard practice involved administration of ASA at a dose of 75 mg once daily, initiated within 24 to 72 h after LVAD implantation depending on postoperative hemostatic status. To assess responsiveness to ASA, platelet function tests were performed using the aspirin-induced platelet inhibition (ASPI) test. Patients classified as ASA-resistant were transitioned to clopidogrel at an initial dose of 75 mg daily. The choice between ASA and clopidogrel was individualized and based on platelet reactivity results, bleeding risk, and drug tolerance. All decisions were made in collaboration with a multidisciplinary LVAD team.

### 2.5. Statistical Analysis

Statistical analyses were performed using R software version 4.5.1 (R Foundation for Statistical Computing, Vienna, Austria). Continuous variables were expressed as median with interquartile ranges or mean ± standard deviation, and compared using the Wilcoxon rank-sum test or Welch *t*-test. Categorical variables were expressed as numbers and percentages, and compared using Fisher’s exact test or Pearson’s chi-squared test. Univariable and multivariable Cox proportional hazard’s models were employed to identify independent predictors of mortality after LVAD implantation. Because the proportional hazards assumption was violated for some variables, we employed a weighted Cox proportional hazards regression approach to obtain unbiased estimates. Variables with a *p*-value < 0.3 in univariable analysis and allowed for the best model fit were entered into the multivariable model. Potential collinearity between explanatory variables was checked by means of Spearman correlation coefficient, Hoeffding’s D statistics, and the Variance Inflation Factor was also assessed. Backward elimination procedure based on the Akaike’s information criterion (AIC) was used to obtain the final model. Validation of the final model was performed using bootstrap method with 1000 replicates, the corrected for optimism c-index was calculated. Hazard ratios (HRs) with 95% confidence intervals (CI) were reported. A *p*-value < 0.05 was considered statistically significant.

## 3. Results

A total of 95 patients with end-stage HF who underwent HeartMate III LVAD implantation were included in the analysis. Among the studied patients, one individual received LVAD as a bridge to recovery, three patients underwent implantation as destination therapy, and the remaining individuals were supported with LVAD as a bridge to transplantation. The median age was 57.9 years (IQR: 47.2–63.8), and 91.6% were male. Ischemic etiology of HF was present in 57.9% of patients. The median observation period was 835 (250–1973) days. During the follow-up, 46 patients (48.4%) died. Over the observation period, device-related infections were observed in 23 patients, bleeding episodes occurred in 48 patients—most of which were classified as mild to moderate—and 11 patients had a stroke or transient ischemic attack (TIA). 21 patients underwent HT during the long-term follow-up.

Before LVAD implantation, 35.8% had a history of nonsustained ventricular tachycardia (nsVT) and 10.5% had experienced electrical storm. Most patients were in NYHA class IV (74.7%) at the time of surgery. Significant differences between survivors and non-survivors were observed in several parameters. Patients in the non-survivors group were significantly older than survivors. Comorbid conditions such as hypertension, type 2 diabetes mellitus, and atrial fibrillation (61.2% vs. 37.0%, *p* = 0.018) were more prevalent among non-survivors. Additionally, the preoperative ALBI score was significantly higher in the non-survivors compared to survivors. No statistically significant differences were observed between groups in echocardiographic parameters, use of cardiac medications (aside from the sodium-glucose cotransporter-2 (SGLT2) inhibitor used), or frequency of device-related complications. The baseline characteristics of survivors and non-survivors were presented in Table 1.

In multivariable weighted Cox analysis, only three baseline variables independently predicted mortality: age (HR 1.04 per year, 95% CI 1.01–1.08; *p* = 0.008), serum creatinine (HR 3.40 per mg/dL, 95% CI 2.05–5.65; *p* < 0.001), and ALBI score (HR 4.98 per unit, 95% CI 2.79–8.89; *p* < 0.001), the corrected c-index was 0.74. Summary of the univariable and multivariable analysis is presented in Table 2.

## 4. Discussion

This single-center retrospective study demonstrated that a simple and inexpensive indicator—the preoperative ALBI score—is strongly associated with increased all-cause mortality following LVAD implantation. Two additional parameters—older age and elevated preoperative creatinine concentration—were also independently associated with worse outcomes, although their prognostic impact was less pronounced.

The ALBI score, originally developed for patients with hepatocellular carcinoma, has become a simple and objective tool for assessing liver function. It integrates two widely available laboratory parameters—serum albumin, reflecting hepatic synthetic function, and bilirubin, associated with the liver’s metabolic and excretory function [7,8]. In patients with advanced HF, hepatic dysfunction is common due to passive congestion, reduced cardiac output, and systemic inflammation [12]. Hypoalbuminemia may result from a combination of decreased hepatic synthesis, malnutrition, and chronic inflammation, and is associated with poor outcomes in various HF populations [13]. In turn, hyperbilirubinemia is a marker of congestive hepatopathy and correlates with central venous pressure and right ventricular dysfunction [14]. Elevated ALBI score has previously been linked with worse prognosis in both acute and chronic HF cohorts [8,9]; however, no prior studies have evaluated their prognostic utility in patients undergoing LVAD implantation. Kawata et al. demonstrated that higher ALBI values at admission were independently associated with increased in-hospital mortality in patients with acute decompensated HF [9]. Similarly, Jurkiewicz et al. reported that ALBI independently predicted long-term survival in elderly HF patients, with a more than fourfold increased risk of death in those with higher ALBI values [8]. Furthermore, the prognostic significance of individual ALBI components has also been investigated in the LVAD population. Kato et al. identified preoperative hypoalbuminemia as an independent predictor of post-LVAD mortality. In the same study, patients with initially low albumin levels who experienced normalization after device implantation had significantly better outcomes than those with persistent hypoalbuminemia during mechanical support [15]. Likewise, Gopal et al. showed that a decline in serum albumin during the first three months after LVAD implantation predicted rehospitalizations and increased mortality [16]. In turn, preoperative bilirubin levels have been linked with adverse outcomes following LVAD implantation. Notably, Shiga et al. reported that elevated total bilirubin prior to pulsatile LVAD placement was an independent predictor of postoperative mortality [17]. In continuous-flow LVAD cohorts, a higher direct bilirubin/total bilirubin ratio at admission strongly predicted early right ventricular failure (AUC ≈ 0.77) [18]. Additionally, preimplant bilirubin elevation has been associated with subsequent hepatic dysfunction after LVAD implantation and reduced one-year survival [19]. Our findings suggest that the combination of both markers of liver function—albumin and bilirubin—may be beneficial in assessing the prognosis after LVAD. ALBI reflects not only hepatic impairment but also the severity of systemic congestion and multiorgan dysfunction in advanced HF. Impaired hepatic synthetic function reduces production of inflammatory proteins and clotting factors, increasing susceptibility to bleeding, infections, and organ failure. Moreover, hepatic congestion exacerbates cholestasis and liver injury post-LVAD, particularly in patients with right ventricular dysfunction or elevated central venous pressure. Impaired liver clearance of toxins and reduced regenerative capacity further compromise postoperative hemodynamic stability [15,18,19].

Although ALBI score has demonstrated good prognostic power in risk stratification of HF patients, further research is required to confirm the effectiveness of this score in analyzed populations. Over the years, many risk-stratification models for HF have been developed; however, they have their limitations and often require complex formula calculations. The INTERMACS profile remains a widely accepted prognostic scale, but it relies on clinical assessment and demonstrates interobserver variability, which can limit reproducibility in profiles in the same patients [20]. In turn, HeartMate risk scores achieve good discrimination in derivation cohorts, but they require numerous variables and have shown mixed utility in contemporary practice [21,22,23]. Another score—MELD-XI, which incorporates only bilirubin and creatinine—has repeatedly predicted post-LVAD outcomes; however, in HF patients creatinine levels change from day to day, depending on hydration status or forced diuresis [24,25,26]. Thus, it appears that the ALBI score has more stable values and reflects the synthetic and cholestatic function of the liver associated with congestion, which represent pathophysiologically important axes in advanced HF and are less susceptible to short-term fluctuations than perioperative creatinine.

Another indicator significantly associated with worse prognosis after LVAD implantation in our study group was higher creatinine level. Numerous studies have documented the adverse prognostic impact of renal dysfunction in LVAD patients [27,28,29,30,31]. In the INTERMACS registry and large meta-analyses, a glomerular filtration rate (GFR) <60 mL/min/1.73 m^2^ was associated with higher risk of death after LVAD implantation [28,29]. Furthermore, while LVADs may improve renal function in the early postoperative period by restoring forward flow and reducing congestion, long-term renal recovery is often incomplete or transient [29,30,31]. Renal dysfunction is a frequent comorbidity in advanced HF and represents a central component of the cardiorenal syndrome. Pathophysiologically, reduced renal perfusion due to low cardiac output, elevated central venous pressure, neurohormonal activation, and systemic inflammation, all act synergistically to worsen GFR in advanced HF [32,33]. Specifically, venous congestion elevates renal venous pressure, reducing renal perfusion pressure and contributing to declining GFR [34]. Simultaneously, persistent congestion and hypoperfusion lead to neurohormonal stimulation—renin–angiotensin system and sympathetic nervous system activation—which contribute to sodium and water retention, oxidative stress, and inflammatory cytokine release, further impairing renal function [20,24]. In the context of LVAD implantation, impaired renal function prior to surgery has been shown to correlate with increased early and late mortality [27,34]. Renal dysfunction may limit the patient’s ability to tolerate perioperative hemodynamic changes and pharmacologic interventions, while also increasing susceptibility to complications such as right heart failure, bleeding, electrolyte disturbances or infection. Additionally, chronic kidney disease has been associated with higher systemic inflammation and poorer tissue perfusion, both of which contribute to impaired postoperative recovery [35,36,37]. This indicates the necessity of close renal monitoring and potential early intervention in patients with reduced renal reserve being considered for mechanical circulatory support (MCS).

Another independent factor associated with a worse prognosis in patients after LVAD implantation in our analysis was older age. While age itself is not a contraindication to MCS, it is increasingly recognized as a surrogate for frailty, comorbidities, and reduced physiologic reserve [38,39]. Elderly patients tend to experience higher rates of adverse events—including bleeding, infection, and right ventricular failure—which may compromise the benefits of LVAD support [38]. Age-related decline in physiological reserve may impair wound healing, immune function, and cardiovascular adaptation to MCS, contributing to worse outcomes [38,39,40,41,42,43]. Data from large registries confirm that advanced age is associated with lower survival after LVAD implantation [40,41,42,43]. In the INTERMACS registry, patients aged ≥75 years experienced 5-year mortality of approximately 66%, compared to roughly 34% in those aged <65 and 54% in those aged 65–75 [40]. Similarly, the MOMENTUM 3 trial identified age as a significant predictor of adverse events and early mortality, particularly among destination-therapy recipients [41,42]. Comparable associations between increasing age and higher early mortality were also reported in the EUROMACS registry, where age was an independent predictor of 90-day mortality following LVAD implantation [43]. Those findings underscore the importance of comprehensive geriatric evaluation, including frailty and functional status assessment, prior to LVAD implantation. In selected elderly patients, alternative management strategies—such as optimal medical therapy or palliative care—may be more appropriate, especially when high frailty burden or limited life expectancy are present.

This study has several limitations that should be emphasized. First, the single-center and retrospective nature of the analysis, relatively small sample size and a high percentage of men may limit the generalizability of the findings to broader patient populations. Furthermore, the cohort size and number of deaths may result in model overfitting or restrict multivariable analysis. Second, although comprehensive clinical and laboratory data were collected, the study may be subject to residual confounding due to unmeasured variables such as frailty, nutritional status and social determinants. Third, the sample size, while adequate for primary endpoint analysis, may have limited power to detect more subtle associations or to fully adjust for multiple covariates in multivariable models. Fourth, although the prognostic value of biomarkers such as ALBI score and serum creatinine was demonstrated, dynamic changes over time were not assessed, and only preoperative baseline values were considered. Furthermore, the sex imbalance may limit the clinical implications of our findings. Finally, cause of mortality were not analyzed separately, which may influence interpretation of survival outcomes. Future multicenter prospective studies are needed to validate the prognostic utility of the ALBI score in LVAD populations and to determine whether perioperative modulation of hepatic function can improve outcomes.

## 5. Conclusions

In summary, our study demonstrated that a higher preoperative ALBI score, elevated serum creatinine, and older patient age were independent predictors of all-cause mortality following HeartMate III LVAD implantation. These findings underscore the clinical value of incorporating readily available laboratory markers and basic demographic variables into preoperative risk stratification. In particular, the ALBI score may serve as a practical and objective tool to identify high-risk patients and guide individualized decision-making prior to LVAD implantation.

## Figures and Tables

**Table 1:** Baseline characteristics of the study population divided into nonsurvival and survival groups.

	All Included Patients N = 95	Nonsurvival N = 46 ^1^	Survival N = 49 ^1^	*p* ^2^
Baseline data				
Age, years	57.9 (47.2–63.8)	53.3 (40.2–61.3)	61.5 (56.3–65.0)	0.006
Male, *n* (%)	87 (91.6%)	42 (91.3%)	45 (91.8%)	0.9
Ischemic etiology of HF, *n* (%)	55 (57.9%)	20 (43.5%)	35 (71.4%)	0.006
nsVT before LVAD, *n* (%)	34 (35.8%)	15 (32.6%)	19 (38.8%)	0.5
nsVT after LVAD, *n* (%)	17 (17.9%)	8 (17.4%)	9 (18.4%)	0.9
Electrical storm in the interview, *n* (%)	10 (10.5%)	4 (8.7%)	6 (12.2%)	0.7
ASA resistance, *n* (%)	26 (27.4%)	12 (26.1%)	14 (28.6%)	0.8
BMI, kg/m^2^	29.0 (24.6–32.7)	27.6 (23.9–32.7)	29.2 (25.2, 33.1)	0.4
NYHA III, *n* (%)	24 (25.3%)	9 (19.6%)	15 (30.6%)	0.2
NYHA IV, *n* (%)	71 (74.7%)	37 (80.4%)	34 (69.4%)	0.2
Comorbidities				
Hypertension, *n* (%)	33 (34.7%)	10 (21.7%)	23 (46.9%)	0.010
Type 2 diabetes, *n* (%)	39 (41.1%)	13 (28.3%)	26 (53.1%)	0.014
AF, *n* (%)	47 (49.5%)	17 (37.0%)	30 (61.2%)	0.018
COPD, *n* (%)	7 (7.4%)	2 (4.3%)	5 (10.2%)	0.4
Stroke after LVAD, *n* (%)	11 (11.6%)	6 (13.0%)	5 (10.2%)	0.7
Device infection, *n* (%)	3 (3.2%)	2 (4.3%)	1 (2.0%)	0.6
Laboratory findings				
WBC, × 10^9^/L	8.8 (7.2–11.3)	8.6 (7.3–9.8)	9.5 (7.2, 12.5)	0.2
Lymphocyte, × 10^9^/L	1.4 (1.1–2.1)	1.6 (1.2–2.2)	1.3 (1.0–1.8)	0.016
RLC, %	16.9 (10.7–21.8)	18.9 (13.6–24.4)	14.4 (8.8–20.2)	0.008
Platelets, × 10^9^/L	198.0 (151.0–250.0)	198.5 (161.0–257.0)	198.0 (134.0–243.0)	0.2
Hemoglobin, mmol/L	13.0 (2.22)	13.3 (2.2)	12.8 (2.3)	0.3
Glucose, mmol/L	106.3 (93.7–136.9)	106.3 (92.8–127.9)	106.3 (95.5–140.6)	0.5
HBA1c, %	5.7 (5.4–6.7)	5.6 (5.4–6.5)	5.9 (5.4–6.7)	0.5
Creatinine on admission, mg/dL	1.4 (1.1–1.7)	1.3 (1.0–1.9)	1.5 (1.1–1.9)	0.3
Creatinine on discharge, mg/dL	0.9 (0.8–1.2)	0.8 (0.8–1.2)	1.0 (0.8–1.6)	0.023
Bilirubin on admission, μmol/L	25.4 (15.2–38.0)	28.0 (19.3–37.3)	20.2 (13.9–38.2)	0.2
Bilirubin on discharge, μmol/L	10.7 (6.9–16.4)	10.7 (7.4–14.8)	11.6 (6.9–20.4)	0.4
Preoperative ALBI score	−2.7 (−2.54–(−1.9)	−2.4 (−2.7)–(−2.1)	−2.1 (−2.42)–(−1.7)	0.006
Albumin on admission, g/L	44.0 (41.0–46.0)	39.5 (35.0–43.0)	40.0 (34.0–45.0)	0.5
Albumin on discharge, g/L	34.0 (31.0–38.0)	36.0 (33.0–39.0)	33.0 (27.0–36.0)	0.005
CRP/bilirubin on discharge	0.4 (0.2–0.95)	0.4 (0.2–1.1)	0.4 (0.2–0.7)	0.3
Urea, µmol/L	11.3 (8.1–16.8)	9.6 (6.2–16.3)	12.9 (9.6–16.8)	0.061
AST, U/L	34.0 (25.0–47.0)	39.5 (27.0–49.0)	31.0 (25.0–41.0)	0.2
ALT, U/L	28.0 (19.0–51.0)	37.5 (21.0–61.0)	23.0 (17.0–34.0)	0.043
Cholesterol, mmol/L	4.3 (3.9–4.9)	3.3 (2.6–4.1)	3.3 (2.6–4.6)	0.4
hs-CRP, mg/L	2.3 (1.9–5.1)	5.9 (1.8–13.5)	4.9 (3.2–9.6)	0.7
Sodium, mmol/L	135.0 (133.0–138.0)	134.5 (131.0–138.0)	135.0 (132.0–139.0)	0.6
LogNTproBNP, pg/mL	8.23 (0.84)	8.07 (0.77)	8.38 (0.88)	0.067
Echocardiographic parameters				
LA, mm	52.4 (9.1)	54.4 (8.9)	50.7 (8.9)	0.085
RVEDd 4CH, mm	41.0 (39.0–44.0)	41.0 (39.0–44.0)	40.0 (38.0–44.0)	0.2
RVEDd M-mode, mm	33.0 (31.0–40.0)	33.0 (30.5–45.0)	33.0 (31.0–39.0)	0.5
TAPSE, mm	14.0 (9.0–17.0)	16.4 (3.9)	16.0 (3.6)	0.6
RVSP, mmHg	43.4 (14.8)	44.5 (32.5–54.0)	40.0 (30.0–53.0)	0.8
LVEDd, mm	77.2 (10.7)	78.4 (11.3)	76.1 (9.9)	0.3
LVEF, %	15.0 (12.0–18.0)	19.5 (15.0–20.0)	20.0 (17.0–20.0)	0.8
Cardiac medication on admission, *n* (%)				
Inotropic support before LVAD implantation, *n* (%)	86 (90.5%)	44 (95.7%)	42 (85.7%)	0.2
B-blockers, *n* (%)	92 (96.8%)	46 (100.0%)	46 (93.9%)	0.2
ACEI, ARB/ARNI *n* (%)	67 (70.5%)	34 (73.9%)	33 (67.3%)	0.5
MRA, *n* (%)	92 (96.8%)	43 (93.5%)	49 (100.0%)	0.11
SGLT2, *n* (%)	33 (34.7%)	21 (45.7%)	12 (24.5%)	0.03
Sildenafil, *n* (%)	91 (95.8%)	44 (95.7%)	47 (95.9%)	0.9
Digoxin, *n* (%)	49 (51.6%)	28 (60.9%)	21 (42.9%)	0.079
Cordarone, *n* (%)	41 (43.2%)	18 (39.1%)	23 (46.9%)	0.4
ASA, *n* (%)	66 (69.5%)	31 (67.4%)	35 (71.4%)	0.7
Clopidogrel, *n* (%)	24 (25.3%)	15 (32.6%)	14 (28.6%)	0.7
VKA, *n* (%)	95 (100.0%)	46 (100)	49 (100)	
ICD, *n* (%)	30 (31.6%)	11 (23.9%)	19 (38.8%)	0.12
CRT-D, *n* (%)	45 (47.4%)	20 (43.5%)	25 (51.0%)	0.5

^1^ Median (Q1, Q3); *n* (%); Mean (SD); ^2^ Wilcoxon rank sum test; Fisher’s exact test; Wilcoxon rank sum exact test; Pearson’s Chi-squared test; Welch Two Sample *t*-test. Abbreviations: ACEI, angiotensin-converting enzyme inhibitor; AF, atrial fibrillation; ALBI, albumin–bilirubin; ALT, alanine aminotransferase; AST, aspartate aminotransferase; ASA, acetylsalicylic acid; ARB, angiotensin receptor blocker; ARNI, angiotensin-receptor neprilysin inhibitors; BMI, body mass index; COPD, chronic obstructive pulmonary disease; CRP, C-reactive protein; CRT-D, cardiac resynchronization therapy-defibrillator; HBA1c, glycated hemoglobin; HF, heart failure; hs-CRP, high-sensitivity C-reactive protein; ICD, implantable cardioverter-defibrillator; LA, left atrium; LVEDd, left ventricular end-diastolic dimension; LVEF, left ventricular ejection fraction; NYHA, New York Heart Association; nsVT, nonsustained ventricular tachycardia; NTproBNP, N-terminal pro-B-type natriuretic peptide; RLC, Relative Lymphocyte Count; RVEDd, right ventricular end-diastolic dimension; RVSP, Right Ventricular Systolic Pressure; TAPSE, Tricuspid Annular Plane Systolic Excursion; SGLT2, sodium-glucose transport protein 2; VKA, Vitamin K Antagonists; WBC, white blood cell; MRA, mineralocorticoid receptor antagonist.

**Table 2:** Univariable and multivariable analyses of factors associated with worse prognosis.

	Univariable Analysis		Multivariable Analysis	
Parameter	HR (95% CI)	*p*	HR (95% CI)	*P*
Age	1.047 (1.018–1.076)	0.001	1.044 (1.011–1.078)	0.008
Ischemic etiology of HF	0.408 (0.219–0.761)	0.005		
Hypertension	1.661 (0.947–2.914)	0.077		
Type 2 diabetes	1.547 (0.882–2.716)	0.13		
AF	1.761 (0.990–3.133)	0.054		
COPD	1.668 (0.660–4.212)	0.3		
RLC	0.982 (0.948–1.018)	0.3		
Bilirubin	1.029 (1.019–1.040)	<0.001		
Creatinine	2.327 (1.351–4.010)	0.002	3.403 (2.050–5.648)	<0.001
ALBI	4.242 (2.506–7.182)	<0.001	4.981 (2.791–8.890)	<0.001
logNTproBNP	1.821 (1.223–2.711)	0.003		

Abbreviations: see Table 1; CI, Confidence Interval; HR, Hazard Ratio.

## Data Availability

The data presented in this study are available on request from the corresponding author. The data are not publicly available due to privacy restrictions related to the rules in our institution.
